# 2318 Augmenting perception through direct electrical stimulation of adult somatosensory cortex

**DOI:** 10.1017/cts.2018.48

**Published:** 2018-11-21

**Authors:** Yohannes Ghenbot, Andrew Richardson, Xilin Liu, Han Hao, Sam DeLuccia, Greg Boyek, Jan Van der Spiegel, Timothy Lucas

## Abstract

OBJECTIVES/SPECIFIC AIMS: Our main objectives are to study sensory encoding in the adult cortex and quantify rodents’ ability to use intracortical microstimulation to guide behavior. METHODS/STUDY POPULATION: Three rats were implanted with unilateral bipolar stimulating electrodes. The electrodes were connected to a wireless neural stimulator housed in the rat’s backpack. The rat’s swim path was tracked by a video camera above the circular pool, and stimulation parameters were updated in real-time based on distance from the platform. Stimulation was delivered as the distance from the platform increased. Stimulation amplitude was determined through behavioral threshold testing, and parameters ranged from 15–75 μA with 100-Hz pulse trains and 0.2-ms pulses. Rats were first challenged with the 4-platform task in which the submerged platform was randomized across 4 possible locations. This dissociated visual cues from the platform location, as rats had knowledge of the 4 possible locations, but had to use stimulation to guide them efficiently. Next, rats were tasked with the more challenging random-platform task. Visual cues were completely dissociated from the platform location by randomizing the platform location across the entire pool. Performance using the neuroprosthetic device was assessed by comparing trials when the device was on (stimulation trial) Versus off (no-stim trial) for the 2 tasks. RESULTS/ANTICIPATED RESULTS: *4-platform task*: Rats visited less potential platform locations when the neuroprosthetic was on Versus off. Rats were also more likely to visit the correct platform location on their first swim trajectory when brain stimulation was delivered. When artificial cues were not available, rats had a greater chance of visiting the platform location from the previous trial. This indicated that rats relied on visuospatial memory without the neuroprosthetic. *Random platform task*: Performance was measured by taking the ratio of the rat’s actual path length to the optimal path length. When the neuroprosthetic was on, rats demonstrated superior performance through a smaller path to length ratio compared with when the device was off. The platform locations of catch trials were matched to a random subset of stimulation trials, permitting a paired sample *t*-test. Both rats had significantly shorter path lengths when the device was on. DISCUSSION/SIGNIFICANCE OF IMPACT: Rodents have excellent navigation skills that have been well studied. They have been shown to rely on multimodal sensory information from visual, olfactory, auditory, and idiothetic cues to navigate through their environment. The importance of these cues depends on both their environmental presence and task relevance. In the original Morris water maze experiment, rats use vision to form a visuospatial map of the platform location for allocentric navigation. Here, we have shown that sensory augmented rats can pick up on novel sensory information delivered through ICMS to efficiently find a hidden platform when visual cues are made irrelevant.Our results have implications for the design of the bi-directional sensorimotor neuroprosthetic. We have demonstrated that mammals can interpret artificial sensory information to guide behavior. Future directions include investigating sensory encoding in other primary sensory areas and downstream targets along the somatosensory neuraxis.

